# *Mycobacterium xenopi* Clinical Relevance and Determinants, the Netherlands

**DOI:** 10.3201/eid1403.061393

**Published:** 2008-03

**Authors:** Jakko van Ingen, Martin J. Boeree, Wiel C.M. de Lange, Wouter Hoefsloot, Saar A. Bendien, Cecile Magis-Escurra, Richard Dekhuijzen, Dick van Soolingen

**Affiliations:** * Radboud University Nijmegen Medical Center, Nijmegen, the Netherlands; † National Institute for Public Health and the Environment, Bilthoven, the Netherlands

**Keywords:** Mycobacterium infections, atypical, Mycobacterium xenopi, Mycobacteria, atypical, research

## Abstract

In the Netherlands, isolation of *Mycobacterium xenopi* is infrequent, and its clinical relevance is often uncertain. To determine clinical relevance and determinants, we retrospectively reviewed medical files of all patients in the Netherlands in whom *M. xenopi* was isolated from January 1999 through March 2005 by using diagnostic criteria for nontuberculous mycobacterial infection published by the American Thoracic Society. We found 49 patients, mostly white men, with an average age of 60 years and pre-existing pulmonary disease; of these patients, 25 (51%) met the diagnostic criteria. Mycobacterial genotype, based on 16S rRNA gene sequencing, was associated with true infection. Most infections were pulmonary, but pleural and spinal infections (spinal in HIV-infected patients) were also noted. Treatment regimens varied in content and duration; some patients were overtreated and some were undertreated.

## Abstract 2

Clinical isolation of *M. xenopi* represents true infection in 51% of cases; genotype is a major determinant.

*Mycobacterium xenopi* was first described by Schwabacher in 1959; it was isolated from skin lesions in a clawed frog and named after the official species designation of the frog, *Xenopus laevis* (*1*). Thereafter, these slow-growing mycobacteria have been recovered from heated water systems in many countries and more recently from natural waters in Finland (*2*). Transmission to humans is believed to originate from the environment, through aerosol inhalation or ingestion. Human-to-human transmission and transmission from animal reservoirs remain controversial because these routes have not been proven by molecular typing (*3*,*4*).

Pulmonary *M. xenopi* infections are most common, but extrapulmonary and disseminated infections have also been recorded (*5*,*6*). A predisposing factor is impaired immunity, either local (e.g., pre-existing pulmonary disease) or systemic (e.g., hematologic malignancy, immunosuppressive medication, or HIV/AIDS) (*5*,*7*).

Its survival in flowing water systems and resistance to common disinfectants enables *M. xenopi* to contaminate laboratory samples and medical devices such as bronchoscopes, thus causing healthcare-acquired (pseudo) infections and laboratory cross-contaminations (*3*,*6*,*8*,*9*). Differentiating true infection from pseudoinfection is of paramount importance because treatment of *M. xenopi* infections is time-consuming and often complicated. The British Thoracic Society (BTS) trial in 2001 established that treatment for pulmonary infections should consist of a 2-year course of rifampin and ethambutol; regimens including macrolides or fluoroquinolones are still being investigated (*10*). The American Thoracic Society (ATS) established general criteria for the diagnosis and treatment of nontuberculous mycobacterial, not specifically *M. xenopi*, infections. The treatment guidelines are similar to those by the BTS, although the ATS guidelines advocate macrolide-containing regimens (*5*).

To assess frequency and clinical relevance of *M. xenopi* isolation and its determinants in the Netherlands, we performed a retrospective case study. We used the ATS diagnostic criteria available during the study period to differentiate true infection from pseudoinfection.

## Methods

To determine clinical relevance, we examined medical records of all patients in the Netherlands from whom *M. xenopi* had been isolated from January 1999 through March 2005. The following variables were extracted from the records: sex, age, predisposing factors, symptoms, chest imaging results, treatment and outcome, and drug susceptibility and status according to the ATS diagnostic criteria (*5*).

Laboratory diagnosis of the isolates was made by the Dutch National Institute of Public Health and the Environment (RIVM) or by a local hospital laboratory. RIVM acts as the national reference laboratory that provides identification, drug-susceptibility testing, and genotyping of mycobacterial isolates for all hospitals and other healthcare institutions in the Netherlands. To identify a mycobacterial isolate, Hain GenoType MTBC line-blot (Hain Lifescience, Nehren, Germany) was used after PCR-based amplification to determine whether an isolate was a member of the *M.* *tuberculosis* complex. If the reaction was negative, an INNO-LiPA MYCOBACTERIA (Innogenetics, Gent, Belgium) reverse hybridization multiple DNA probe assay was used to differentiate between the more common species of nontuberculous mycobacteria, including *M. xenopi* (*11*). Before 2004, 16S rRNA gene sequence analysis was performed, after ruling out membership in the *M. tuberculosis* complex, by using the AccuProbe MTB DNA probe kit (GenProbe, San Diego, CA, USA). The result was compared with the RIVM and BLAST (National Center for Biotechnology Information, www.ncbi.nlm.nih.gov) 16S rRNA gene sequence databases (*12*).

On the basis of the results at position 90 in the 151-bp hypervariable region of the 16S gene, 2 *M. xenopi* genotypes were discerned; a C at position 90 distinguished *M. xenopi* I and a T distinguished *M. xenopi* II. Retrospectively, the 16S rRNA genes of all *M. xenopi* isolates at RIVM were sequenced and assigned to their respective genotypes.

Susceptibility testing was performed by using an agar dilution method (*13*). Drugs in the susceptibility testing panel were isoniazid, rifampin, ethambutol, streptomycin, cycloserine, prothionamide, amikacin, ciprofloxacin, clofazimine, clarithromycin, and rifabutin. The Pearson χ^2^ test was used for statistical correlations. The local ethics committee approved the study.

## Results

We found 49 patients with nontuberculous mycobacterial infection (Table 1); of these, 25 (51%) met the ATS diagnostic criteria. Isolates from 46 patients were identified to the genotype level, I or II. Sequencing failed for 2, and 1 was unavailable at RIVM. *M. xenopi* I was found for 28 patients, *M. xenopi* II for 13, and mixed (types I and II) for 5. Isolation of type II was significantly associated with fulfillment of the ATS criteria compared with isolation of type I only (77% vs 39%; odds ratio [OR] 5.1, 95% confidence interval [CI] 1.2–23.0, p = 0.025). When we defined *M. xenopi* II cultures as “involving *M. xenopi* II,” and thus included the mixed cultures, the correlation increased in significance (OR 5.4, 95% CI 1.4–20.8, p = 0.011).

**Table 1:** Baseline population characteristics of 49 patients with nontuberculous mycobacterial infection, the Netherlands, January 1999 through March 2005*

Characteristic	ATS+ (n = 25)	ATS– (n = 24)	Total (%)
Demographics			
Male sex	19	18	37 (76)
Mean age, y	60	60	60
Dutch origin	24	20	44 (90)
Concurrent and predisposing conditions			
Pre-existing pulmonary disease	21	18	39 (80)
Chronic obstructive pulmonary disease	17	14	31 (63)
Lung cancer	1	3	4 (8)
Prior tuberculosis	0	2	2 (4)
Recurrent pulmonary infection†	5	2	7 (14)
Bronchiectasis	2	4	6 (12)
Smoker, current/ past	15/ 6	11/ 3	35 (71)
Alcohol abuse	2	3	5 (10)
High-dose steroid use‡	3	5	8 (16)
HIV infection	2	5	7 (14)
Mean CD4 count in HIV-infected patients, cells/mL	226	126	159
Hematologic malignancy	0	1	1 (2)
Otherwise impaired immunity§	2	1	3 (6)
Signs and symptoms			
Productive cough	21	20	41 (84)
Hemoptysis	5	4	9 (18)
Dyspnea	14	9	23 (47)
Fever	11	6	17 (35)
Weight loss	12	7	19 (39)
Malaise	16	10	26 (53)
Chest radiographic abnormalities			
Infiltrate	15	12	27 (55)
Cavity	12¶	3	15 (31)
Pleural thickening	3	4	7 (14)
Emphysema	9	9	18 (37)
Space-occupying lesion	1	3	4 (8)

*ATS+, American Thoracic Society diagnostic criteria for nontuberculous mycobacterialinfection met; ATS–, ATS diagnostic criteria for nontuberculous mycobacterial infection not met. †>3 requiring treatment in 6 months before primary *Mycobacteria xenopi* culture. ‡**>**15 mg prednisone/day for >3 months before primary *M. xenopi* culture. §Diabetes mellitus, cisplatinum chemotherapy, anorexia nervosa (all n = 1). ¶Significant association (odds ratio 14.3, 95% confidence interval 2.7–75.6, p = 0.001).

Clinical signs and symptoms varied widely and were not associated with fulfillment of the ATS diagnostic criteria (Table 1). Chest radiographs were taken for all patients, except for 2 who had spinal infection (Table 1). Cavitation was the only radiographic finding significantly associated with fulfillment of the ATS diagnostic criteria (OR 14.3, 95% CI 2.7–75.6, p = 0.001). Results of additional computed tomography scanning, performed for 27 patients, were not associated with fulfillment of the ATS diagnostic criteria (data not shown).

We found 4 cases of extrapulmonary disease, 2 cases of pleural *M. xenopi* infection, and 2 cases of spondylodiscitis (in HIV–co-infected patients). The pleural infections were diagnosed by biopsy of pleural tissue for 1 patient and repeated culture of pleural fluid for the other, after chest radiograph demonstrated pleural thickening and fluid collection. The spinal infections were diagnosed by bone biopsy. In the pleural and bone biopsy specimens, granulomatous lesions with central necrosis were observed.

For most patients, *M. xenopi* was first isolated from sputum (51%), bronchoalveolar lavage fluid (35%), or lung biopsy sample (4%). Remaining isolates were from bone biopsy samples (4%), pleural fluid (2%), pleural biopsy samples (2%), and stool samples (2%). Acid-fast bacilli were detected with direct microscopy of primary samples for 39% of patients. An acid-fast bacilli–positive primary sample, regardless of its nature, was significantly associated with fulfillment of the ATS diagnostic criteria (OR 8.2, 95% CI 2.1–31.6, p<0.001).

Treatment was started for 25 of 49 patients, of whom 19 met the ATS diagnostic criteria. Therapy consisted of medication for 21 patients, surgery for 2, or both for 2. Surgery consisted of lobectomy, pulmonary wedge resection, Clagett pleurostomy, and vertebral surgery with psoas muscle abscess drainage. Medication regimens varied widely but generally included rifampin, isoniazid, ethambutol, clarithromycin, ciprofloxacin, and pyrazinamide in various 3- to 4-drug combinations. Duration of therapy varied between 5 days and 2.5 years, with a mean duration of 9 months. Macrolides were included in regimens for 58% and quinolones for 37% of the patients who met the ATS diagnostic criteria and received drug treatment.

Antimycobacterial treatment cured 11 (58%) patients who met the ATS diagnostic criteria: 7 with *M. xenopi* II, 2 with *M. xenopi* I, and 2 with *M. xenopi* I and II. We defined cure as resolution of symptoms and negative cultures after finishing treatment, until the end of our study period (range 0–60 months, median 25 months). Treatment failure, defined as protracted culture positivity for *M. xenopi* during and after adequate treatment, was noted for 4 (21%). Four other patients died. Treatment failure or death was not associated with genotype, susceptibility pattern, predisposing conditions, or radiographic imaging results.

Although they fulfilled the ATS diagnostic criteria, 4 patients did not receive treatment. Of these, 1 recovered spontaneously; 2 remained positive for acid-fast bacilli, culture, or both throughout the study period; and 1 died. These outcomes were not associated with specific genotypes or patient factors.

Susceptibility testing was performed for 47 isolates from 42 patients. For 5 patients, cultures failed to grow for testing; for 2 others, cultures were not available. Results for isoniazid, rifampin, and ethambutol are shown in Table 2. Isolates were susceptible to all other compounds tested. Susceptibility testing of follow-up cultures was performed for 5 patients*. M. xenopi* bacteria in 2 patients treated with rifampin became resistant to rifampin and to ethambutol in 1 patient. For 9 patients, susceptibility testing results influenced the treatment regimens, mostly by inclusion or exclusion of rifampin and ethambutol or by adding a quinolone or macrolide agent.

**Table 2:** Baseline in vitro susceptibility of 47 primary isolates from 42 patients with nontuberculous mycobacterial infection, the Netherlands, January 1999 through March 2005

Susceptibility	Drug, no. (%), MIC		
	Isoniazid	Rifampin	Ethambutol
Susceptible	9 (21), MIC 0.2 mg/L	29 (69), MIC <1mg/L	5 (12), MIC 5 mg/L
Intermediate	32 (76), MIC 0.5–1.0 mg/L		11 (26), MIC 10 mg/L
Resistant	1 (2), MIC >1 mg/L	13 (31), MIC >1 mg/L	26 (62), MIC >10 mg/L

For 6 patients with cavitation visible on chest radiograph, of whom 4 met the ATS diagnostic criteria, fungi were cultured simultaneously (*Aspergillus fumigatus* from 4, *A. flavus* from 1, and *Scedosporium apiospermum* from 1). Antifungal treatment was initiated for 4 patients, which meant true nontuberculous mycobacteria infection was left untreated for 2.

Four patients who had received antimycobacterial treatment for *M. xenopi* infection before (mean duration 8 months) had relapses; the mean interval between discontinuation of drug treatment and relapse was 28 months (range 12–39). We found no evidence of geographic clustering, which suggests nosocomial transmission or a pseudo-outbreak. The number of new isolates per year remained steady at ≈8 per year. At least 5 patients were treated in preventive isolation for 2–15 days until *M. tuberculosis* complex infection was excluded by PCR.

## Discussion

Clinical relevance of *M. xenopi* isolation, defined by fulfillment of the ATS diagnostic criteria, was likely in 51% of patients; mycobacterial genotype II was a major determinant. To our knowledge, this phenomenon and its causal mechanisms have not been described. If further evidence emerges, 16S rRNA gene sequencing may become a relevant addition to the diagnostic algorithm of *M. xenopi* infection.

The ATS diagnostic criteria are designed for *M. avium*, *M. kansasii,* and *M. abscessus* infections, although the authors state “there is no reason to believe these criteria would not be applicable to other species” (*5*). Because the BTS statement focuses on management rather than specific diagnostic criteria (*14*), the ATS diagnostic criteria are recommended for the clinical setting. Of the main ATS diagnostic components, 2 were each significantly associated with true infection in our study (cavitary lesions on chest radiograph and acid-fast bacilli on primary samples), thereby supporting the ATS criteria.

The ATS diagnostic criteria, however, have 1 limitation. Patients with pre-existing cavitary lesions are likely to have respiratory symptoms; they meet the radiologic criteria and are more likely to harbor mycobacteria in the cavity, which are not necessarily responsible for their symptoms and cavity formation. Cavity characteristics cannot reliably predict the cavity’s origin or pathogenesis (*15*). The uncertainty is compounded when fungi are cultured simultaneously, which suggests matching requirements for in vivo success of these microorganisms or selective impaired immunity. Determining which organism causes disease in the patient is difficult.

The undertreatment and overtreatment that we noted indicates a relative lack of knowledge in physicians, mainly those specializing in pulmonary conditions, concerning nontuberculous mycobacteria infections. Unnecessary drug treatment could harm the patient in terms of adverse effects and costs (*16*), and undertreatment of patients who fulfill the diagnostic criteria is potentially harmful to the patients’ health.

The baseline characteristics of our study group are similar to those in studies of nontuberculous mycobacteria patients in societies with low HIV prevalence (*5*,*10*). The North American series included more HIV-infected patients, which lowered the mean age of patients in these studies (*17*,*18*). Pre-existing pulmonary diseases are major predisposing factors and may be causally associated with isolation of *M. xenopi*. However, *M. xenopi* may have been isolated more often because physicians were more focused on mycobacterial cultures for this category of patients.

Minor predisposing factors were HIV infection or other causes of impaired immunity, which, in addition to the causal relationship, probably reflect the low prevalence of HIV infection compared with chronic pulmonary disease in the Netherlands. Also, because most HIV-infected patients receive highly active antiretroviral therapy, fewer cases of severe HIV immunosuppression and its co-infections are seen. HIV infection predisposes patients to extrapulmonary *M. xenopi* infection, especially spinal infection (*6*,*19*). Those with HIV-associated spinal *M. xenopi* infection had rising CD4 counts after starting or changing highly active antiretroviral therapy regimens (Table 1). Possibly, their *M. xenopi* infection was an expression of immune reconstitution inflammatory syndrome (*20*). We found no previous reports of pleural infections.

Although the treatment regimens recorded in our study were not in accordance with the current standards of the BTS (*14*) and ATS (*5*), cure rates were high. Although partly the result of the restricted definition of “cure” resulting from our research methods, this finding does bring the validity of the ATS diagnostic criteria into question. Despite meeting these criteria, some patients might have cleared *M. xenopi* infection without treatment. This possibility is endorsed by the spontaneous recovery of a minority of patients who met the ATS criteria but were not treated. Alternatively, the regimen of 24 months of rifampin and ethambutol advised by the BTS may not be better than similar regimens of shorter duration. The addition of macrolides and quinolones to therapy regimens might also account for the high cure rates after relatively short treatment durations.

Susceptibility testing results were similar to those published previously (*21*), but their value in clinical practice is uncertain. Interpretation of the laboratory results is difficult because of discrepancies between in vitro susceptibility and in vivo response to treatment (*5*,*21*). In our study, results rarely influenced treatment regimens; when they did, choice of regimen was controversial and not in accordance with ATS and BTS guidelines (*5*,*14*). Increasing use of PCR to rule out *M. tuberculosis* infection can be valuable for preventing or shortening patient isolation.

In conclusion, clinical isolation of *M. xenopi* was relevant for 51% of the patients; mycobacterial genotype was a major determinant. Currently, the ATS diagnostic criteria are the best tool for determining clinical relevance. We strongly recommend increased awareness of these diagnostic criteria and management guidelines by ATS and BTS.
